# Novel Method for Differentiating Histological Types of Gastric Adenocarcinoma by Using Confocal Raman Microspectroscopy

**DOI:** 10.1371/journal.pone.0159829

**Published:** 2016-07-29

**Authors:** Chih-Wei Hsu, Chia-Chi Huang, Jeng-Horng Sheu, Chia-Wen Lin, Lien-Fu Lin, Jong-Shiaw Jin, Lai-Kwan Chau, Wenlung Chen

**Affiliations:** 1 Division of General Surgery, Department of Surgery, Tungs’ Taichung MetroHarbor Hospital, Taichung City, Taiwan; 2 Department of Applied Chemistry, National Chiayi University, Chia-Yi, Taiwan; 3 Center of Nano Bio-detection, National Chung Cheng University, Chia-Yi, Taiwan; 4 Department of Applied Cosmetology, HungKuang University, No.34, Zhongqi Rd., Shalu Township, Taichung County, Taiwan; 5 Division of Gastroenterology, Department of Internal Medicine, Tungs’ Taichung MetroHarbor Hospital, Taichung City, Taiwan; 6 Department of Pathology, Tungs’ Taichung MetroHarbor Hospital, Taichung City, Taiwan; Oita University Faculty of Medicine, JAPAN

## Abstract

Gastric adenocarcinoma, a single heterogeneous disease with multiple epidemiological and histopathological characteristics, accounts for approximately 10% of cancers worldwide. It is categorized into four histological types: papillary adenocarcinoma (PAC), tubular adenocarcinoma (TAC), mucinous adenocarcinoma (MAC), and signet ring cell adenocarcinoma (SRC). Effective differentiation of the four types of adenocarcinoma will greatly improve the treatment of gastric adenocarcinoma to increase its five-year survival rate. We reported here the differentiation of the four histological types of gastric adenocarcinoma from the molecularly structural viewpoint of confocal Raman microspectroscopy. In total, 79 patients underwent laparoscopic or open radical gastrectomy during 2008–2011: 21 for signet ring cell carcinoma, 21 for tubular adenocarcinoma, 14 for papillary adenocarcinoma, 6 for mucinous carcinoma, and 17 for normal gastric mucosas obtained from patients underwent operation for other benign lesions. Clinical data were retrospectively reviewed from medical charts, and Raman data were processed and analyzed by using principal component analysis (PCA) and linear discriminant analysis (LDA). Two-dimensional plots of PCA and LDA clearly demonstrated that the four histological types of gastric adenocarcinoma could be differentiated, and confocal Raman microspectroscopy provides potentially a rapid and effective method for differentiating SRC and MAC from TAC or PAC.

## Introduction

Gastric adenocarcinoma is one of the common diseases and accounts for approximately 10% of cancers worldwide [1]. It has been considered a single heterogeneous disease with multiple epidemiological and histopathological characteristics [2]. The World Health Organization (WHO) categorizes, based on histological classification, gastric adenocarcinoma into four types: papillary adenocarcinoma (PAC), tubular adenocarcinoma (TAC), mucinous adenocarcinoma (MAC), and signet ring cell adenocarcinoma (SRC) [3]. According to the degree of glandular formation, TAC is classified as well-, moderately- or poorly-differentiated, while PAC is graded as well-differentiated and SRC as poorly-differentiated [4]. However, the Japanese classification system categorizes gastric adenocarcinomas into two groups: differentiated and undifferentiated. The differentiated group consists of well-differentiated, moderately-differentiated TAC and PAC. The undifferentiated group consists of poorly differentiated adenocarcinoma and SRC. MAC is regarded as either a differentiated or undifferentiated type [5]. Classification of gastric adenocarcinoma remains ambiguous, and whether MAC and SRC are the result of poor prognostic factor is in argument. Some researchers reported that both MAC and SRC are due to poor prognosis [6–11]; however, some investigators disagreed to that [12–14]. For example, previous studies have demonstrated that the poor prognosis of MAC is due to the advanced stage rather than histological type [4,15–17]. Both SRC and MAC are mucin-producing cancers. SRC does not form glandular tubules but accumulates mucin in the cytoplasm [18], whereas mucin is drained from cancer cells in MAC [18]. Therefore, SRC is diagnosed when adenocarcinoma is a predominant component with more than half of isolated tumor cells containing intracellular mucin [16,19]. In contrast, MAC is diagnosed when more than half of the tumor area contains extracellular mucin pools [16,19]. The 5-year survival rates of SRC and MAC were as low as 15.9% and 19.4%, respectively [20]. How to increase the survival rates of SRC and MAC is an urgent issue. The survival rate of gastric adenocarcinoma is closely related to its early diagnosis and treatment. Hence, developing an efficient tool to detect this cancer at early stage is of great importance.

Tis or T1a, an early stage cancer, is limited to mucosa and may be a candidate for endoscopic mucosal resection [21]. However, the lymph node metastasis of mucosal SRC is more frequent and early SRC should not be treated using endoscopic resection [22]. SRC exhibits more infiltrative tumor growth and has a higher incidence of lymphatic spread and peritoneal seeding [3,6,14]. Some studies have suggested that SRC patients benefit from total gastrectomy with extended lymphadenectomy [13,23,24]. Therefore, differentiating SRC and MAC preoperatively or intraoperatively is crucial.

The histopathological examination based on the morphology of biopsy specimens has been usually used as a standard method for diagnosing gastric adenocarcinomas. The conventional method, however, suffers from several shortcomings including invasive process, requiring time-consuming tissue processing (generally 2–3 days), morphological masquerade of tissues, and lack of precision due to the pathologist’s visual reading of the specimens [25–28]. For example, SRC cells are difficult to identify because of high mucin content and can be confused with muciphages, which are mucoprotein-containing macrophages [27]. Some muciphages have low mucin content and can be confused with lymphocytes [27]. In addition, some early MACs are submucosal tumor-like lesions covered by surrounding normal mucosa because of MAC production of abundant extracellular mucin and extensive growth in the submucosal layer [28]. This could render a timely and accurate diagnosis difficult.

Recently Raman spectroscopic methods have shown great potential in biomedical applications [29–38]. Raman spectroscopy is a vibrational spectroscopy. Raman signals arise from inelastic scattering when photons impinging on a sample transfer energy to or from a molecular vibrational mode. Because the energy transfer is unique for every molecule, Raman spectroscopy provides a lot of molecular information and is powerful in characterizing molecular structure. Varieties of Raman techniques such as FT-Raman, resonance Raman, surface-enhanced Raman scattering, tip-enhanced Raman, coherence anti-stoke Raman, and confocal Raman microscope have been developed in biological and biomedical applications [25,29–31]. Each Raman technique exhibits its unique preference in a certain usage. Confocal Raman microscope offers decisive advantages in contrast enhancement, rejection of stray light, and discrimination of a well defined spatial region in a complex multiphase specimen. Confocal microscope provides an efficient way to obtain interference-free Raman spectra. The optical microscope focusing laser point onto a diffraction-limit spot on the specimen improves significantly in the lateral resolution and depth discrimination. Coupling with the advances in the CCD detector, confocal Raman microcope provides a noninvasive, interference-free, less sample preparative, and spatially resolving method for studying the molecular composition of gastric tumors. Raman microscope has been successfully applied in intraoperatively cancer-related study such as brain tissue classification [32,33], breast cancer margin evaluation [34–36], axillary lymph nodes assessment [37], and head and neck squamous cell carcinoma investigation [38]. However, there remains lack of information using confocal Raman microscopy in the histological types, classified by the WHO, of gastric adenocarcinoma. In this report, we investigated the different histological types of gastric adenocarcinoma by using confocal Raman microspectroscopy and demonstrated a more rapid and effective method for differential diagnosis of different histological types of gastric adenocarcinoma.

## Materials and Methods

### Patients and tissues

In total, 79 patients were enrolled. Tests were conducted on 62 patients with gastric adenocarcinoma and 17 patients with normal gastric tissues. All 62 patients with gastric adenocarcinoma underwent laparoscopic or open radical gastrectomy and were categorized into four histological types during 2008–2011 at Tungs’ Taichung MetroHarbor Hospital: 21 for SRC, 21 for TAC, 14 for PAC, and 6 for MAC; normal gastric tissues were collected from 17 patients who underwent operation for other benign lesions. The specimens were preserved in wax, and 3-μm-thick sections were prepared. Slides containing the sections were dewaxed with hexane for 5 min, ethanol for 3 min, and methanol for 1 min, and then dried with N_2_ before Raman measurement.

The pathological diagnosis of these cases was reviewed by at least two experienced pathologists. Clinical data were retrospectively reviewed from medical charts.

### Ethics statement

This study was approved by the Institutional Review Board of Tungs’ Taichung MetroHarbor Hospital (approval number: 100006). All patients were informed of the involved procedures, and they provided written consent before the collection of all specimens and clinical information.

### Raman Measurement

Confocal Raman spectra of gastric adenocarcinoma and normal gastric mucosa were obtained by using a confocal laser micro-Raman system (MploRA, Horiba Jobin-Yvon, France) with CCD detector. All Raman measurements were performed at room temperature. Confocal Raman microspectroscopy provides a platform for acquiring detailed Raman spectra from small volume of specimens. A Raman spectrometer with 532-nm laser excitation coupled with a confocal microscope (Olympus BX41, NA = 0.9) with 100x objective lens was used. The solid-state diode laser with 8 mW was used as an excitation source. The excitation light beam was directly shining on the surface of specimen, and the back-scattered light was collected, passed through the entrance slit (100 μm wide), dispersed by a diffraction grating (1200 grooves/mm), and detected by an air-cooled CCD detector. The exposure time for each spot was 100 s (integration time 1 s, accumulation: 100 scan), and 5–10 spots depending on the sample size were chosen for each specimen. The area was primarily diagnosed as cancer abundant area by pathologist. Raman spectra were produced over the Raman shift 500–3100 cm^−1^.

### Statistical analysis: Principal component analysis and linear discriminant analysis

Spectral data were processed and analyzed by accessing the ArrayTrack—Supporting Toxicogenomic Research at the U.S. Food and Drug Administration National Center for Toxicological Research [39]. Each spectrum from 500 to 3100 nm was divided into 1022 segments and analyzed using principal component analysis (PCA) and linear discriminant analysis (LDA) to distinguish histological types of gastric adenocarcinoma. The first two calculated principal components (PC1 and PC2), which contained most of the information, were plotted against each other for visualization. LDA with 20-fold cross validations was used to examine the accuracy, sensitivity, and specificity of differential diagnosis through confocal Raman microspectroscopy. LDA used a combination of independent variables to maximize the separation among the different histological groups of gastric adenocarcinoma and normal gastric mucosa. The analyses and scatter plots were performed using the SPSS 22.0 (SPSS, Chicago, IL) software package.

PCA is a multivariate technique used to classify and to reduce the dimensionality of the spectral data. Orthogonal linear combinations to transform the original data into uncorrelated variables are termed PCs. LDA is another data reduction technique. PCA uses the most information from the original data and LDA maximizes the intergroup differences and minimizes the intragroup differences. Therefore the eigenvectors of PCA and LDA are different. [40, 41] We analyzed the Raman spectra of all 79 patients and 62 adenocarcinoma patients with PCA. Because the samples of these two training were different, the PCs of these two test were also different. Receiver operating characteristic (ROC) curves were generated by successively changing the thresholds to determine correct and incorrect classifications for all subjects. The threshold of the sensitivity and specificity was defined as the maximum sum of the sensitivity and specificity (Youden index) [40–42].

## Results

### Raman spectroscopic investigation

**Fig 1:**
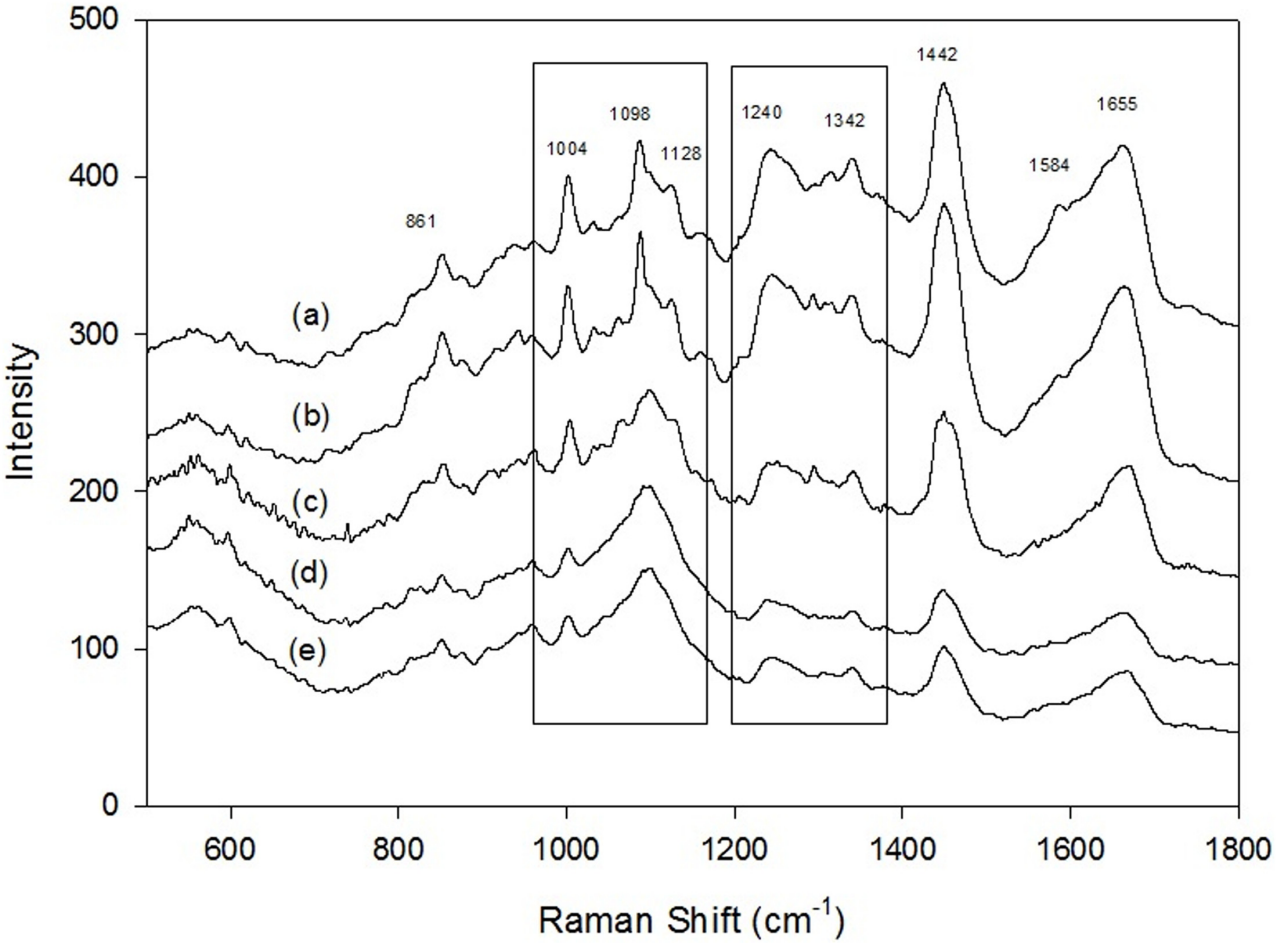
Raman spectra, in the range of 550–1800 cm^-1^, of gastric adenocarcinomas. **(a)** normal gastric mucosa; (b) signet ring cell adenocarcinoma; (c) mucinous adenocarcinoma; (d) tubular adenocarcinoma; (e) papillary adenocarcinoma.

In total, 79 patients were enrolled. They underwent laparoscopic or open radical gastrectomy because the preoperative diagnosis was gastric adenocarcinoma. Seventeen of them who underwent operation for other benign lesions were enrolled, and their normal gastric mucosa was preserved for this study. The postoperative pathological reports included 21 patients with SRC, 21 with tubular adenocarcinoma, 14 with papillary adenocarcinoma, and 6 with MAC. Each specimen was detected 5–10 times by confocal Raman microspectroscopy. Fig 1 demonstrated Raman spectra of normal gastric mucosa (Fig 1a), SRC (Fig 1b), MCA (Fig 1c), TAC (Fig 1d), and PAC (Fig 1e). Raman signals at 861, 1004, 1098–1128, 1240, 1342, 1442, 1584, and 1655 cm^−1^ were respectively assigned to the vibrational modes of C–C stretching, C–C symmetric stretching, C–N stretching, C–N stretching and N–H bending, CH_3_CH_2_ wagging, CH_2_ and CH_3_ bending, C = C bending, and C = O stretching [43]. Significant spectral difference among the four types of adenocarcinoma and the normal one was apparent in two segments, 1098–1128 cm^−1^ and 1240–1342 cm^−1^.

### Principal component analysis and linear discriminant analysis

**Fig 2:**
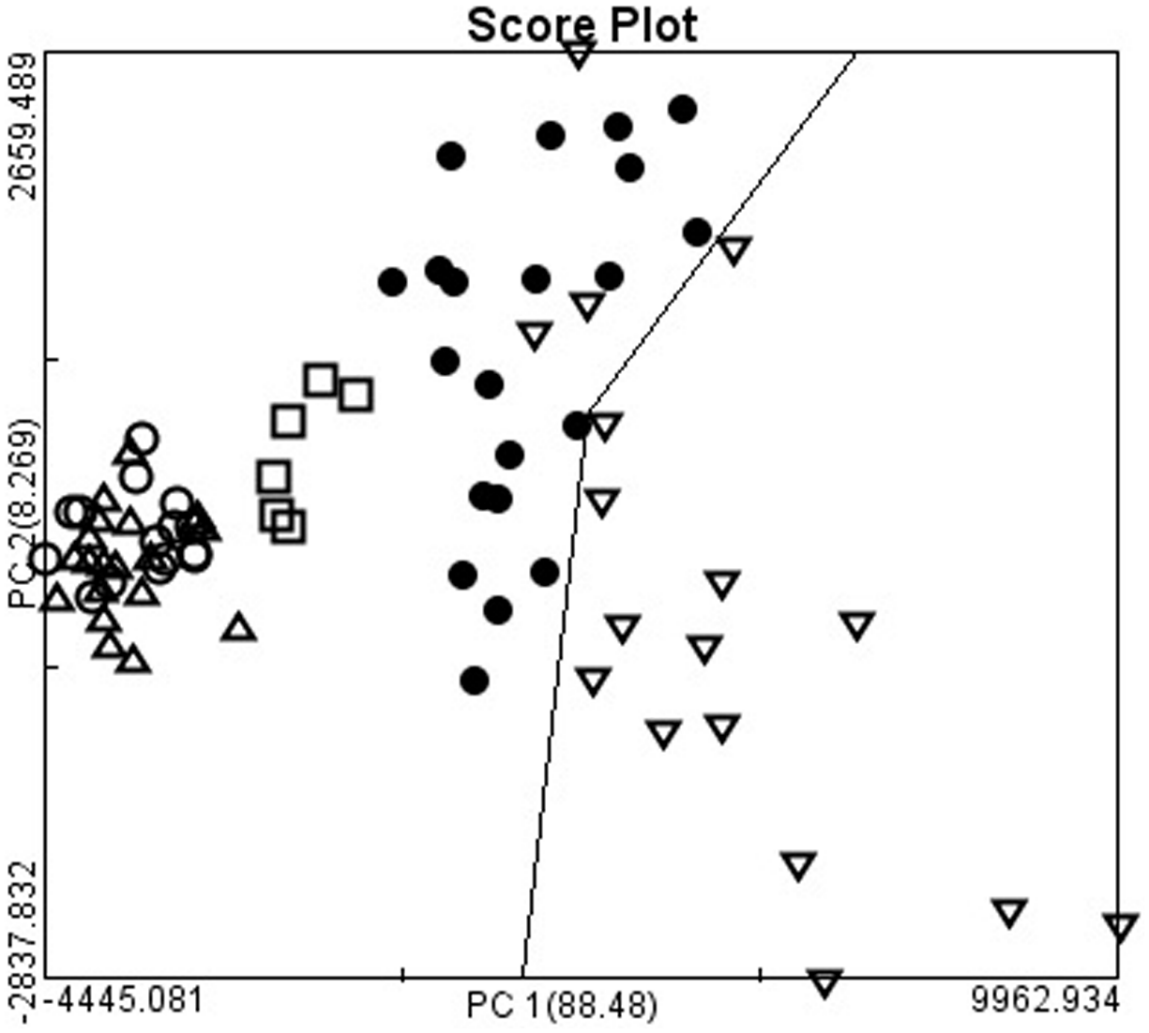
The two-dimensional plots of gastric adenocarcinomas and normal gastric mucosa. The first two calculated principal components (PC1 and PC2), which contained most of the information, were plotted against each other for visualization. The PC1 accounting for the largest Raman spectra variance was 88.48%, and PC2 was 8.26%. The two-dimensional plots showed the separation of gastric adenocarcinoma from normal gastric mucosa (▽: normal gastric mucosa; ●: signet ring cell adenocarcinoma; □: mucinous adenocarcinoma; Δ: tubular tubular adenocarcinoma; ○: papillary adenocarcinoma).

**Fig 3:**
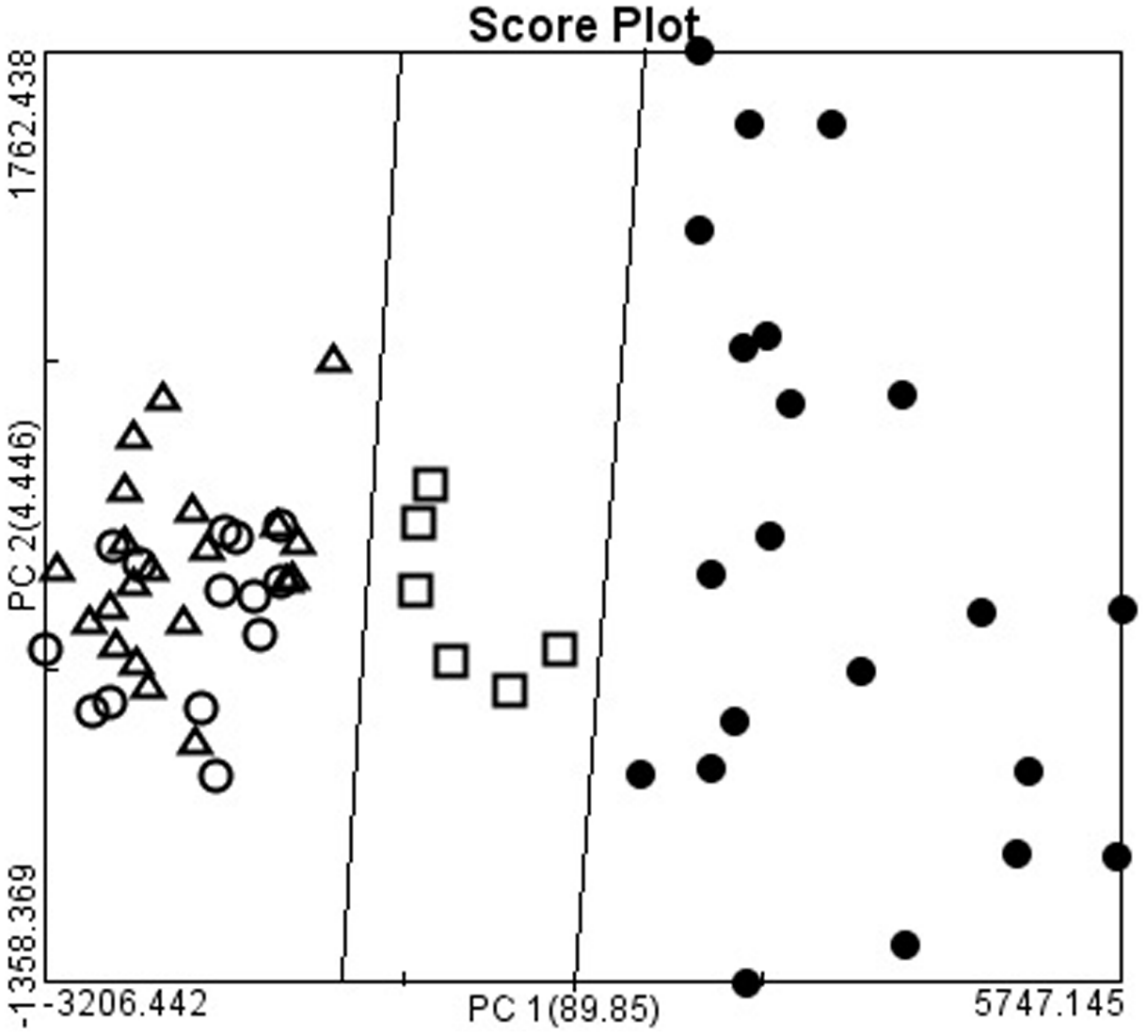
The two-dimensional plots of gastric adenocarcinomas. The first two calculated principal components (PC1 and PC2), which contained most of the information, were plotted against each other for visualization. The PC1 accounting for the largest Raman spectra variance was 89.85%, and PC2 was 4.45%. The two-dimensional plots showed the separation of signet ring cell adenocarcinoma, mucinous adenocarcinoma, from and tubular carcinoma and papillary adenocarcinoma (●: signet ring cell adenocarcinoma; □: mucinous adenocarcinoma; Δ: tubular adenocarcinoma; ○: papillary adenocarcinoma).

**Fig 4:**
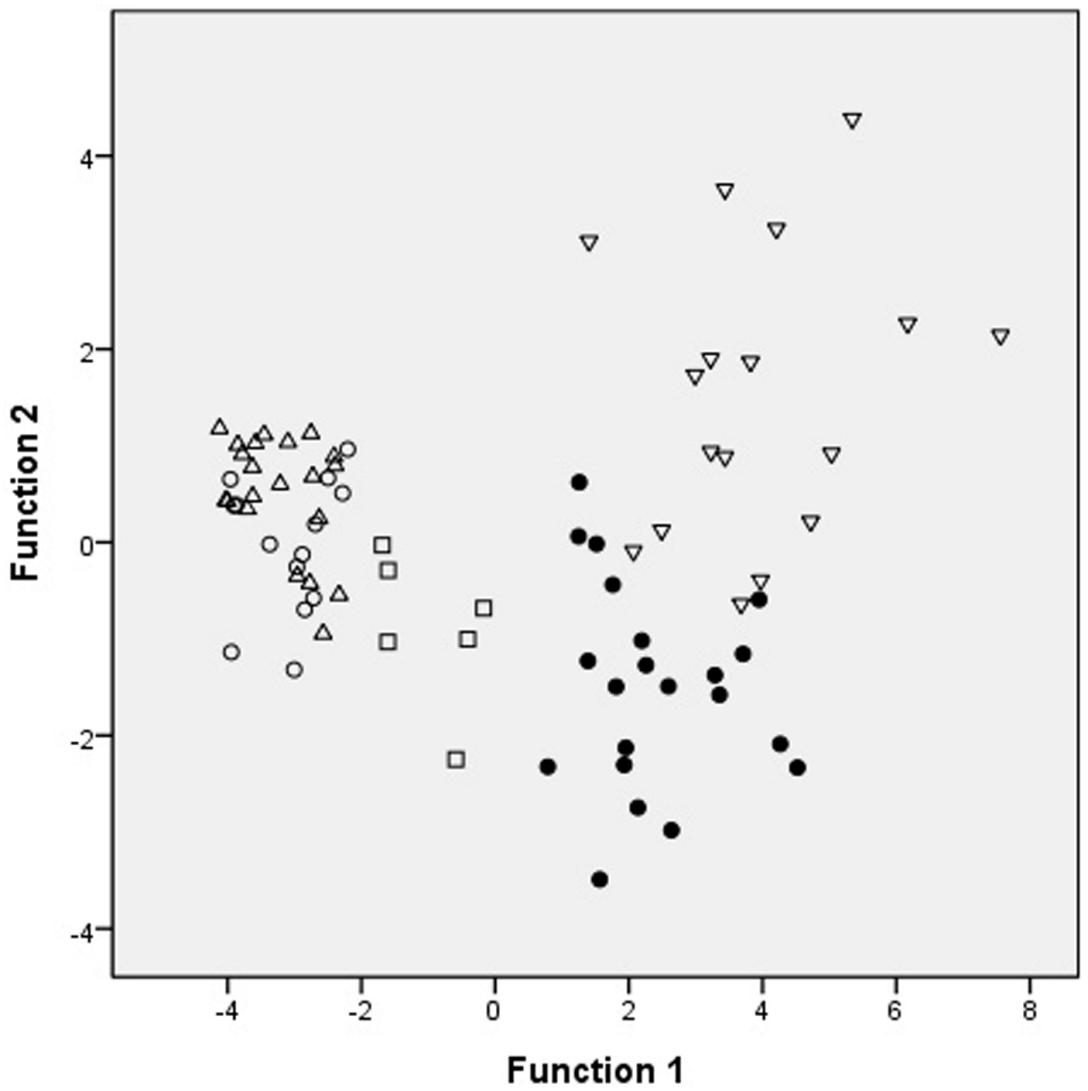
The two-dimensional plots of gastric adenocarcinomas and normal gastric mucosa. Scatter plots of the first and second important discriminant function plotted against one another. The two-dimensional plots showed the separation of gastric adenocarcinoma from normal gastric mucosa (▽: normal gastric mucosa; ●: signet ring cell adenocarcinoma; □: mucinous adenocarcinoma; Δ: tubular tubular adenocarcinoma; ○: papillary adenocarcinoma).

**Fig 5:**
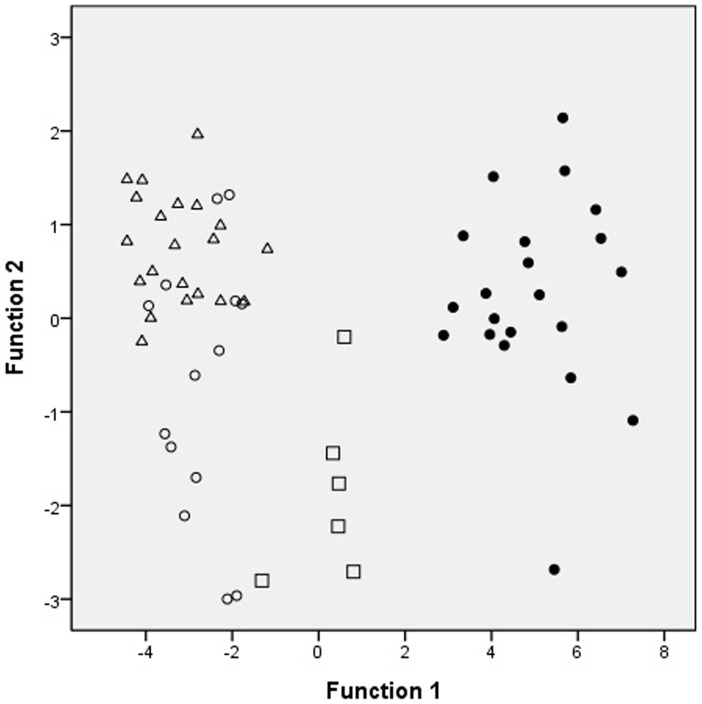
The two-dimensional plots of gastric adenocarcinomas. Scatter plots of the first and second important discriminant function plotted against one another. The two-dimensional plots showed the separation of signet ring cell adenocarcinoma, mucinous adenocarcinoma, from and tubular carcinoma and papillary adenocarcinoma (●: signet ring cell adenocarcinoma; □: mucinous adenocarcinoma; Δ: tubular adenocarcinoma; ○: papillary adenocarcinoma).

The four types of adenocarcinoma and normal gastric mucosa were further analyzed by using PCA. Each point was the average spectrum of one patient; the results are shown in Fig 2. Gastric adenocarcinoma was effectively differentiated from the normal gastric mucosa by using PCA. The results of two-dimensional PCA on different histological types of gastric adenocarcinoma were shown in Fig 3. The PC1 accounting for the widest Raman spectra variance was 89.85%, and PC2 was 4.45%. SRC and MAC were effectively differentiated from TAC or PAC. However, no significant difference was observed between TAC and PAC. The results were verified using 20-fold cross validation. The sensitivity of SRC and MAC was 100% and 98.21%, respectively; the specificity was 100% and 100%, and the accuracy was 100% and 98.39%, respectively (Table 1). We also differentiated the four types of adenocarcinoma and normal gastric mucosa by using LDA scatter plots and the results were shown in Fig 4. Gastric adenocarcinoma was effectively differentiated from the normal gastric mucosa by using LDA scatter plots. The results of LDA scatter plots on the different histological types of gastric adenocarcinoma were shown in Fig 5. Each of the samples clearly was classified into the correct type of gastric adenocarcinoma as classified by the WHO.

**Table 1:** Performance report of sample classification by using 20-fold cross validation.

Metric	Signet Ring Cell	Mucinous
Accuracy	100.00%	98.39%
Sensitivity	100.00%	98.21%
Specificity	100.00%	100.00%
Positive predictivity value	100.00%	100.00%
Negative predictivity value	100.00%	85.71%
False positive rate	0.00%	0.00%
False negative rate	0.00%	1.79%
Matthew's correlation coefficient	1.00	0.92
Area under ROC curve	1.00	0.99

## Discussion

Raman spectroscopy is a powerful tool to investigate the molecular structure of interests. Difference in the molecular composition and conformation of a compound will be correctly revealed by the vibrational mode in the Raman spectra. Hence, we employed confocal Raman microscope to differentiate the different types of gastric adenocarcinoma. Adenocarcinoma originates from the epithelial cells of the mucosal layer. The biochemical and biomolecular characteristics of the different histological types of gastric adenocarcinoma are believed to be different, which would result in demonstrating different Raman spectra. The vibrational stretching of gastric adenocarcinoma (Fig 1) at 861, 1004, 1098–1128, 1240, 1342, 1442, 1584, and 1655 cm^−1^ were respectively assigned to categories of proline, phenylalanine, phospholipids, amide III, collagen, phospholipids, phenylalanine, and amide I [43]. In the band of 1098 cm^−1^, the spectra of MAC and SRC split into a major peak of 1088 cm^−1^ and a smaller peak of 1110 cm^−1^; however, these peaks were not observed in TAC or PAC. These results indicate that phospholipids in the different adenocarcinomas are different. In addition, the intensity ratio of the peak of 1240/1342 cm^−1^ was different. In MAC and SRC, the intensity of the peak of 1240 cm^−1^ was slightly higher than that of the peak of 1342 cm^−1^. However, the intensity of the peak of 1240 cm^−1^ was significantly higher than that of the peak of 1342 cm^−1^ in TAC and PAC. Furthermore, an additional peak of 1310 cm^−1^ was observed in the spectra of MAC. The proportion of amide III and collagen in these three types of gastric adenocarcinomas was also different. Different biochemical and biomolecular characteristics presented in the different types of gastric adenocarcinoma can be explicitly distinguished by the confocal Raman microscope. A study using electron microscopy [20] found that SRCs lacked in free ribosomes but were abundant in rough endoplasmic reticula (RER), lysosomes, mucus granules, and Golgi complexes, meaning that SRCs had a strong capability of protein and mucus synthesis. In addition, few microvilli were present on the surface of desmosomes and the gap junction of the cell membranes, suggesting a decrease in adhesive ability and easy detachability among cancer cells. In contrast to SRCs, MACs were abundant in free ribosomes and had scattered RER and few Golgi complexes and lysosomes. These findings suggested that MACs had stronger adhesive ability than SRCs and released more sulfuric acid mucopolysaccharide. The mucin content of heavily glycosylated oligosaccharide side chains was associated with the protein backbone [4].

Yasuda and coworkers [28] used intraoperative frozen sections to determine the distal margin in gastric adenocarcinoma, achieving a sensitivity, specificity, and accuracy of 100%; the proximal margin exhibited a sensitivity, specificity, and accuracy of 67%, 100%, and 93%, respectively. The falsely deemed negative margins on frozen sections were due to overlooked signet ring cells. Therefore, confocal Raman microspectroscopy offers a potential method for differentiating SRCs and MACs from TAC or PAC. The Raman microspectroscopic method demonstrates the possibility of in situ measurement as combined with endoscopy [44,45]. In addition, Raman measurement only takes a few minutes coupling with the advantage of without tissue pretreatment such as immunohistochemistry staining. However, some limitations should be overcome by promoting Raman spectroscopy a general diagnostic tool for gastric adenocarcinomas. The compositions of proteins or other molecules specific to gastric cancer tissues are complexes, and interpreting the spectrum of each histological type of gastric adenocarcinoma requires well-trained investigator. In addition, gastric cancer tissues are always accompanied by chronic inflammation [46]. This would result in structural and molecular changes which would complicate spectral interpretation. Since Confocal Raman microspectroscopy is limited to detect focal lesions and because of tumor heterogeneity, a combined endoscopy or endoscopic ultrasound in situ study will help to further evaluate the characteristics of gastric tissues and that may perform measurements intraoperatively.

In conclusion, confocal Raman microspectroscopy provides potentially a quick and effective method for differentiating SRC and MAC from TAC or PAC preoperatively or intraoperatively to facilitate effective and immediate decision making.

## Supporting Information

S1 File Raw Data. The original spectra and corresponding histological subtypes.
(XLSX)
